# Insulin Signaling in Bone Marrow Adipocytes

**DOI:** 10.1007/s11914-019-00552-8

**Published:** 2019-11-20

**Authors:** Michaela Tencerova, Meshail Okla, Moustapha Kassem

**Affiliations:** 1 Department of Molecular Endocrinology, KMEB, University of Southern Denmark and Odense University Hospital, 5000 Odense C, Denmark; 2 Present Address: Department of Molecular Physiology of Bone, Institute of Physiology, Czech Academy of Sciences, 142 20 Prague 4, Czech Republic; 3 Department of Community Health Sciences, College of Applied Medical Sciences, King Saud University, Riyadh, Saudi Arabia; 4 Stem Cell Unit, Department of Anatomy, College of Medicine, King Saud University, Riyadh, Saudi Arabia; 5 Department of Cellular and Molecular Medicine, The Novo Nordisk Foundation Center for Stem Cell Biology (DanStem), Panum Institute, University of Copenhagen, Copenhagen, Denmark

**Keywords:** Bone marrow adipose tissue, Marrow adiposity, Insulin signaling, Bone marrow mesenchymal stem cells

## Abstract

**Purpose of Review:**

The goal of this review is to discuss the role of insulin signaling in bone marrow adipocyte formation, metabolic function, and its contribution to cellular senescence in relation to metabolic bone diseases.

**Recent Findings:**

Insulin signaling is an evolutionally conserved signaling pathway that plays a critical role in the regulation of metabolism and longevity. Bone is an insulin-responsive organ that plays a role in whole body energy metabolism. Metabolic disturbances associated with obesity and type 2 diabetes increase a risk of fragility fractures along with increased bone marrow adiposity. In obesity, there is impaired insulin signaling in peripheral tissues leading to insulin resistance. However, insulin signaling is maintained in bone marrow microenvironment leading to hypermetabolic state of bone marrow stromal (skeletal) stem cells associated with accelerated senescence and accumulation of bone marrow adipocytes in obesity.

**Summary:**

This review summarizes current findings on insulin signaling in bone marrow adipocytes and bone marrow stromal (skeletal) stem cells and its importance for bone and fat metabolism. Moreover, it points out to the existence of differences between bone marrow and peripheral fat metabolism which may be relevant for developing therapeutic strategies for treatment of metabolic bone diseases.

## Introduction

Bone marrow adipose tissue (BMAT) comprises approximately 8% of total fat mass and thus representing a significant fat depot in adult humans with a role in bone homeostasis and whole body energy metabolism [1]. BMAT is more predominant in the appendicular than in axial skeleton [2]. The fatty acid composition of BMAT varies considerably based on its anatomical location, but tibia BMAT was found to have a fatty acid profile that resembles white and classical brown adipose tissues [3]. In the post-natal organism, BMAT originates from progenitor cells that are distinct from peripheral adipose tissues. BMAT is thought to be derived from bone marrow stromal (skeletal, mesenchymal) stem cells (BMSC) present within the bone marrow stroma and that are capable for differentiation, in addition to adipocytes, into osteoblasts and chondrocytes [1]. Currently, there is no consensus regarding the phenotype of BMAT progenitors but a number of markers have been proposed to identify adipocyte progenitors within the bone marrow, including osterix [4, 5], Prx1 and Nestin1 (reviewed in [6]), leptin receptor [7], Rankl [8], Znf423 [9••], and Hoxa11 [10].

BMAT plays a role in lipid storage, skeletal remodeling, and hematopoietic regulation but the mechanisms mediating and integrating these diverse functions have not yet been fully delineated [3]. In addition, BMAT is recognized as an endocrine organ producing local and systemic factors including adiponectin, dipeptidyl peptidase 4 (DPP4), legumain (LGMN), secreted frizzled-related protein 1 (sFRP-1), and delta-like 1 (DLK1) (also known as preadipocyte factor 1 (Pref1)) [9••, 11••, 12–14]. BMAT undergoes pathologic changes during aging and in a number of diseases [1, 2, 4]; e.g., it expands in anorexia nervosa, states of estrogen deficiency, glucocorticoid excess, and growth hormone deficiency [2, 11••].

Another evidence about the role of BMAT to participate in regulating whole body energy metabolism is its ability to respond to insulin [15], to activate Sirt1, a key cellular energy sensor, and to induce a thermogenic gene program [16]. BMAT may contribute to systemic glucose and fatty acid clearance [3]. In addition, BMAT responds to insulin-sensitizing anti-diabetic medications such as thiazolidinedione (TZD) drugs, PPARγ agonists [4, 15, 17].

**Fig. 1:**
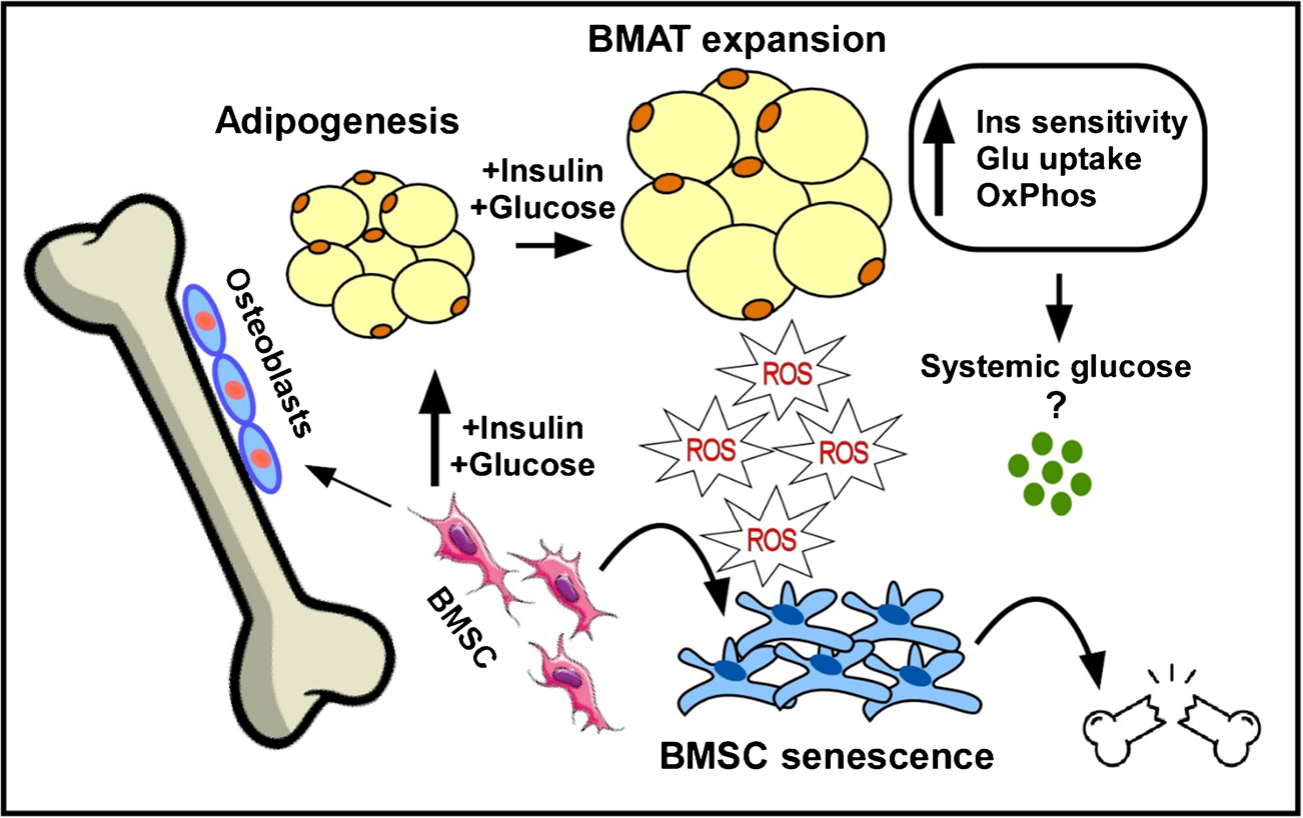
A working model for the role of insulin signaling in bone marrow adipocyte development, metabolic function and cellular senescence. Active insulin signaling, as in the state of obesity, increases the pro-adipogenic potential of bone marrow stromal stem cells (BMSC) leading to bone marrow adipose tissue (BMAT) expansion, which is associated with enhanced insulin sensitivity, glucose uptake, and oxidative phosphorylation. This metabolic phenotype of BMSC in obesity results in increased ROS production, which might lead to creation of senescent bone marrow microenvironment and stem cell exhaustion contributing to bone fragility in metabolic bone diseases

In the current review, we will discuss the role of insulin signaling in bone marrow adipocyte formation, metabolic functions, and its contribution to cellular senescence (Fig. 1). We will also summarize common factors involved in the regulation of BMSC differentiation fate into BMAT with a special reference to obesity and type 2 diabetes (T2D).

## Insulin Signaling in Bone Marrow Adipocytes

### Insulin and Insulin Receptor

Bone marrow adipocytes express insulin receptors [18••, 19] and insulin signaling is essential for BMAT formation and function (reviewed in [17]). Ablation of insulin receptor decreases BMAT volume in distal tibia as a result of a reduction in adipocyte size, but not in number [20]. In genetically reconstituted insulin receptor knockout mice (that are euglycemic as a result of human insulin receptor transgene expression in the pancreas, liver, and brain), BMAT is suppressed and whole tibial mRNA level of aP2, a marker of mature adipocytes, is also reduced compared with wild-type tibias [21]. On the other hand, insulin signaling activation in HFD-fed mice associates with elevations in adipogenic genes and expansion in BMAT [18••]. Interestingly, BMAT expansion accompanying obesity has no adverse effects on insulin sensitivity in marrow adipocytes, unlike peripheral white adipocytes, which manifested impaired insulin sensitivity [18••]. Similar to obese mice, activation of insulin signaling in obese humans stimulates a pro-adipogenic differentiation of BMSC [18••, 22••] (depicted in Fig. 1). Adults with morbid obesity and T2D, who have high serum insulin levels, exhibited higher total BMAT at the lumbar spine and femoral metaphysis compared with those without diabetes [23]. Thus, current literature suggests that alterations in insulin receptor function or insulin level regulate BMAT formation.

### Insulin Receptor Substances 1 and 2

Insulin responses are mediated largely through signaling substrates insulin receptor substance 1 (IRS-1) and insulin receptor substance 2 (IRS-2) [24] and a number of studies have demonstrated that IRS-1 and IRS-2 play a regulatory role in BMAT formation. IRS-1 and IRS-2 are expressed in BMAT and regardless of BMAT expansion, their gene expression was not reduced by HFD in obese mice as observed in visceral fat [18••]. In fact, IRS-1 gene expression was induced by prolonged HFD (20 weeks) [18••]. The specific role of IRS-1 and IRS-2 in BMAT formation *in vivo* has not been studied. A recent *in vitro* study, however, showed that IRS-1 negatively regulates rodent BMSC adipogenesis as overexpression of IRS-1 decreased the gene expression of adipogenic markers *Cebpβ* and *Pparγ*, while IRS-1 deficiency upregulated *Cebpβ* and *Pparγ*. The mechanism by which IRS-1 regulates rat BMSC adipogenesis in this study was mediated partially by PI3K-AKT and MEK-ERK pathways [25]. Contrary to IRS-1, IRS-2 positively regulates adipogenesis in human BMSC as *Irs-2* expression was induced during adipogenesis, while IRS-2 deficiency repressed adipogenesis and led to downregulation of *Cebpα* and *Pparγ*. In addition, targeting IRS-2 in human BMSC by miR-431 negatively regulated adipogenesis and decreased the gene expression of *Cebpα* and *Pparγ* [24]. IRS-2 can compensate for IRS-1 dysfunctions as BMSC of IRS-1-deficient mice showed induced IRS-2 expression, which was reported to be mediated by miR-33 downregulation [26].

### Insulin-Like Growth Factor-1

Insulin-like growth factor-1 (IGF-1) plays an important role in the regulation of bone marrow adiposity [24, 27]. IGF-1 mediates biological effects mostly through binding to IGF-1 receptor and with less affinity to insulin receptor [28, 29]. There is a negative correlation between plasma IGF-1 and BMAT formation [30]. In subjects with a severely reduced caloric intake, dysregulation of the growth hormone-IGF-1 axis and low leptin levels are associated with increased BMAT formation [31]. In obese women who underwent bariatric surgery, the increase in serum IGF-1 levels is associated with declines in BMAT volume. In addition, in a cross-sectional study, obese premenopausal women with higher levels of IGF-1 have lower vertebral marrow fat content independent of age and BMI [32]. Likewise, growth hormone replacement in rats that underwent hypophysectomy exhibit reduced BMAT [33]. Moreover, BMSC obtained from mice with decreased serum IGF-1, due to knockdown of IGF-1 production by the liver or knockout of its binding proteins, revealed a greater adipogenic potential compared with controls [34]. *In vivo* analysis of the bone marrow of IGF-1 mutant mice showed elevated levels of adipogenic markers [34].

### IGF-Binding Protein 4

IGF-binding proteins (IGFBPs) are regulators of tissue levels and functions of IGF. Among IGFBPs, IGF-binding protein 4 (IGFBP4) is required for bone marrow adipocyte formation. *In vitro* cultures of primary BMSC exhibit a strong induction of *Igfbp4* gene expression with a 15-fold increase during early stage of adipocyte differentiation [27]. Moreover, primary BMSC from male and female IGFBP4^−/−^ mice exhibit reduced adipogenesis in vitro [27]. These experiments suggest that IGFBP4 is involved in bone marrow adipocyte formation.

## Factors Interacting with Insulin in Regulating BMSC Differentiation Fate to Adipocytes

### Glucose

Recent studies have demonstrated that the skeleton contributes to systemic nutrient clearance and is the second highest contributing tissue to systemic glucose clearance [3]. A positive relationship between serum glucose and marrow adiposity has been reported [17]. Change in glucose metabolism is an important determinant of marrow fat modulations after gastric bypass surgery and improvement in the glycemic control is associated with reduced marrow fat content [35]. The effect of glucose status on human BMSC adipocyte differentiation has been tested by incubating cells with sera obtained from women with T2D that promote adipocyte differentiation and result in a significant increase in the expression of adipogenic genes (*aP2*, *Lpl*, and *Pparγ*) and an increase in lipid accumulation [36]. In addition, glycated hemoglobin (HbA1c) is positively correlated with vertebral BMAT content of L1–L3 in diabetic women. Diabetic women with hemoglobin A1C levels > 7%, exhibit higher vertebral BMAT content of L1–L3 compared with patients with HbA1c levels ≤ 7% [37]. Moreover, a high level of blood glucose inhibits the proliferation and migration of BMSC and promotes marrow adipocyte formation but not osteoblastogenesis [15]. These studies demonstrate that BMAT formation is affected by glycemic status and glycemic control; however, further studies are needed to determine whether these effects are independent of insulin action.

### Receptor for Advanced Glycation End Products

RAGE is the receptor for advanced glycation end products (AGEs), the products of non-enzymatic glycation and oxidation of proteins that form in hyperglycemic conditions (reviewed in [38]). RAGE signaling pathway plays a role in the pathogenesis of diabetes complications (reviewed in [38]); however, due to its ability to bind multiple ligands and its role in perpetuating and amplifying inflammatory responses, RAGE activation is involved in several inflammatory diseases, such as arteriosclerosis, Alzheimer’s disease, arthritis, acute respiratory failure, and sepsis (reviewed in [39]). In T2D model (db/db mice) and T1D model (streptozotocin (STZ)-injected mice), the abundance of endogenous BMSC is reduced as determined by colony-forming unit assay [40]. RAGE-KO mice are protected from STZ-induced BMSC reduction and RAGE-KO BMSC exhibit enhanced adipocyte differentiation evidenced by formation of a higher number of Oil Red O–stained mature adipocytes and higher expression levels of adipocyte marker genes, e.g., fatty acid-binding protein 4 (*Fabp-4*), *Pparγ*, and *Cebpα* compared with wild-type BMSC [40]. Furthermore, RAGE-KO BMSC show a greater potential to differentiate into osteoblasts evidenced by increased expression of osteoblast gene markers, including *Alpl*, *Osx*, *Bglp*, *Runx2*, *Bmp2*, and *Bmp4* [40]. Based on the above, RAGE signaling may mediate the effect of insulin and glucose in diabetic patients on BMSC properties, and thus, this pathway represents a potential target in the treatment of diabetic bone disease [40].

### Parathyroid Hormone

Diabetic bone disease increases the risk of postmenopausal and age-related osteoporotic fractures [41]. Intermittent parathyroid hormone (PTH) treatment stimulates bone formation and reduces osteoporotic bone loss and osteoporotic fracture risk [41, 42]. Stimulation of type I PTH/PTHrP receptor signaling enhances osteoblast and reduces adipocyte differentiation [42]. In leptin receptor-labeled bone marrow stromal population, PTH treatment shifts cell fate from adipocytic to osteoblastic lineage as evidenced by decreased expression levels of the adipocyte markers such as *Cebpβ*, *Pparγ*, and *Zfp467*. Comparably, genetic deletion of the PTH1R in BMSC progenitors resulted in high bone marrow adiposity and low bone mass in rodents [43]. In ovariectomized mice, PTH treatment suppressed the expansion of the BMAT [42]. Therefore, bone marrow adipocytes are responsive to PTH and the positive effects of PTH treatment on bone formation may be mediated by a shift in the differentiation fate of BMSC from adipocytes toward osteoblasts [43]. PTH treatment in mice with T1D increases trabecular bone mass, mineral apposition, and osteoblast surfaces in addition to suppression of osteoblast apoptosis [41], but in contrast to the abovementioned studies, the positive effects of PTH on bone mass are not accompanied by a reduction in diabetes-induced BMAT accumulation [41].

### Glucagon-Like Peptide-1

Glucagon-like peptide-1 (GLP-1) is an intestinal hormone that plays an essential role in the regulation of glucose homeostasis. In human adipose–derived stromal stem cells, GLP-1 stimulated osteoblastic cell differentiation and suppressed adipogenesis [44]. In these cells, inhibiting ERK reversed the anti-adipogenic effect of GLP-1 [44]. In human BMSC, Exendin-4, a stable GLP-1 gut hormone analogue currently used for the treatment of T2D, promotes both adipocytic and osteoblastic differentiations probably due to increased number of committed progenitors. On the other hand, Exendin-4 induces lipolysis in mature adipocytes and does not affect osteoblast metabolic activity [45]. In hindlimb-unloading-induced bone loss rat model, Exendin-4 treatment enhances bone formation and it decreases adipocyte number in the bone marrow. Also, Exendin-4 promotes osteoblastic differentiation and inhibits adipocytic differentiation in rat BMSC via regulation PKA/β-catenin and PKA/PI3K/AKT/GSK3b signaling [46]. Although GLP-1 may regulate BMSC cell fate and BMAT formation, the effect of GLP-1 on obesity- or diabetes-associated BMAT expansion remains to be determined.

### Secretory Factors within Bone Marrow Microenvironment

Several extracellular factors present within BMSC niche play a role in lineage allocation to adipocytes versus osteoblasts. Our group has identified a number of factors secreted within bone marrow microenvironment that participate in regulation of BMAT formation including DLK1, sFRP-1, and LGMN [12–14] that regulate the differentiation fate of BMSC into osteoblasts versus adipocytes. Among these, DLK1 seems to play a role in regulating insulin effects on the skeleton. DLK1 is co-localized with insulin within the secretory granules of pancreatic β-cells. Under-caboxylated osteocalcin (Glu-OCN), a hormone produced by osteoblastic cells, stimulates pancreatic insulin secretion and also production of DLK1. Interestingly, DLK1 antagonizes the effects of insulin on osteoblast production of Glu-OCN and thus represents a mechanism preventing OCN-induced hypoglycemia [47]. These studies demonstrate the close association of secreted factors present in bone marrow and skeleton microenvironment and whole body energy metabolism.

## Insulin Signaling in Bone Marrow Adipocytes in Relation to Obesity

Insulin exerts anabolic effect on the bone metabolism and it has a critical role in the regulation of skeletal development and bone integrity [48]. Insulin signaling represents a key metabolic pathway important for the bioenergetic demand of bone cells [49–53]. Our group has recently examined the effects of obesity on BMAT and its role in regulating skeletal energy demands. We have observed a unique metabolic phenotype of BMAT in obese mice and obese humans that exhibit enhanced insulin signaling, which was opposite to what we observed in peripheral adipose tissue [18••, 22••]. In more details, we identified a pro-adipogenic potential of BMSC in mice fed HFD and in obese humans driven by increased insulin signaling associated with enhanced oxidative phosphorylation, leading to BMAT expansion. We have also observed enrichment in a unique BMSC population with high expression of insulin receptor (IR+) in obese subjects [22••]. Interestingly, our study suggests that in murine and human obesity, bone marrow microenvironment does not exhibit insulin resistance phenotype. While this seems at variance with previously published data that employed animal models, most of the studies have employed insulin receptor (INSR)-deficient mice as a model for insulin resistance [49, 50, 51••]. This discrepancy may be explained by the biological differences between INSR-deficient mice, which is a suitable model for insulin deficiency taking place in long-standing T2D and obese subjects who exhibited insulin resistance phenotype but no manifest diabetes. Further, murine studies did not examine the presence of intrinsic changes in BMSC, as the observed insulin resistance may have been related to microenvironmental factors. On the other hand, the study by Wei et al. [51••] corroborates our findings as it demonstrates that mice with enhanced insulin signaling in bone are protected from the severe systemic insulin resistance phenotype. Our study demonstrates that maintenance of insulin responsiveness in BMSC of obese subjects is “a protective mechanism” allowing fat storage in bone marrow, when peripheral tissues manifest impaired insulin signaling (Table 1 summarizes the major findings of these studies and illustrating similarities and differences between studies). We think that tissue-specific responses to insulin are highly relevant for the bone field with respect to understanding the pathophysiology of obesity and T2D-associated bone disease.

**Table 1:** Overview illustrating similarities and differences between animal and human studies investigating the role of insulin signaling in bone homeostasis

Study	Reference	Model	Mouse strain	Gender and age	Diet/Intervention	Insulin phenotype in bone tissue	Immunohisto-/in situ hybridization of INSR
Cell Ferron et al. [50]	PMID: 20655470	Mouse	Col1a1/Insr KO (Insr_obs_ KO); Ocn/Insr_obs_ KO; Esp/ Insr_obs_ KO or Fox01/Insr_obs_ KO	Male (8–12 weeks)	Chow and high-fat diet (58% fat kcal) 6 weeks	–	–
Cell, Fulzele et al. [49]	PMID: 20655471	Mouse	OC-IR KO (Ob-delta IR)	Male (3–6 weeks)	Chow diet	–	Osteoblast
JCI, Wei et al. [51••]	PMID: 24642469	Mouse	Col1a1/Insr KO and Col1a1/Insr Tg	Male	Chow and high-fat diet (58% fat kcal) 3 months	Insulin resistant in bone in WT and Col1a1/Insr KO mice;	–
JBMR, Tencerova et al. [18••]	PMID: 29444341	Mouse	C57BL/6J	Male (12–20 weeks)	High-fat diet (60% fat kcal) 3–5 months	Insulin sensitive in BM-MSC	–
Tencerova et al. [22••]	PMID: 31091445	Human	–	Male (age 19–50)	–	Insulin sensitive in BM-MSC	Bone marrow adipocyte

## Insulin Signaling and Metabolic Programming of BMSC

Stem cells existing in states of commitment and differentiation have specific bioenergetic needs that determine their functions [54, 55]. Bone marrow consists of heterogeneous population of BMSC with different lineage commitments [56, 57]. Our group has recently examined whether metabolic programming of BMSC is upstream of lineage commitment [22] (Tencerova et al., 2019 *Bone Research*, accepted 10.1038/s41413-019-0076-5).

Stem cells get energy supply from either glycolysis or oxidative phosphorylation (OxPhos) and they differ in the choice of energy substrate depending on whether they are in the growth or differentiation stage. Pluripotent embryonic stem cells prefer anabolic glycolysis, which is also the preferred metabolic process of rapidly proliferating cells [58, 59]. Hematopoietic progenitor cells exhibit differentiation dependent use of glycolysis or OxPhos [60, 61]. Osteoblast lineage cells employ both oxidative and glycolytic metabolic pathways in undifferentiated state, but during osteoblast differentiation, glycolysis is the preferred energy source [62]. On the other hand, a pre-adipocytic embryonic murine cell line 3T3-L1 employs OxPhos during adipocyte differentiation [62]. Thus, commitment to either osteoblasts or adipocytes is associated with a characteristic bioenergetic profile that is maintained during differentiation. We have recently observed that human BMSC and committed murine BMSC progenitors exhibit similar bioenergetic phenotype [22••] (Tencerova et al., *Bone Research*, accepted 10.1038/s41413-019-0076-5). Employing high-throughput technologies including RNAseq and metabolomics, we found that committed murine adipocyte progenitors (named BMSC^adipo^) and osteoblast progenitors (named BMSC^osteo^) [57] exhibit a distinct metabolic program that is dependent on insulin signaling with higher oxidative phosphorylation in BMSC^adipo^ compared with preferable glycolysis in BMSC^osteo^. Our findings demonstrate that the BMSC exhibit cellular responses to exogenous metabolic signals, which regulate their differentiation fate and the expansion of osteoblast versus adipocyte progenitor populations dependent on the prevalent metabolic environment. Future studies are needed to investigate the contribution of bioenergetic properties of BMSC to whole body energy homeostasis.

## Insulin Signaling and Senescence in Bone Marrow—a Dual Role of this Pathway

Metabolic pathways play a critical role in aging [63]. Insulin signaling belongs to nutrient sensing pathways that are important not only for energy metabolism but also for regulation of cellular senescence in different cell types [64].

Insulin signaling has been shown to regulate mitochondrial function [65, 66], which produces the most of cellular energy in form of ATP but this process is also accompanied with formation of intermediates such as reactive oxygen species (ROS) that play a role as a second messenger. Recent study has demonstrated that ROS increases insulin sensitivity via oxidative modification of the insulin receptor (autophosphorylation) or inactivation of protein tyrosine phosphatases, including PTEN and PTP1B [67]. Thus, this oxidative challenge suggests activation of cellular adaptation via modulation of insulin action and antioxidant system [68–70]. In physiological condition, ROS is also required for adipocyte differentiation, which is controlled by insulin signaling [71]. On the other hand, chronic exposure to ROS in obesity and diabetes is associated with insulin resistance [72] as ROS is also a trigger for cell damage, inflammation, and senescence. Insulin signaling regulates mitochondrial function and biogenesis by inhibiting FOXO1, which tunes redox signaling by maintaining NAD+/NADH ratio for activation of SIRT1/PGC1α important for normal mitochondrial function [73, 74]. SIRT1 deacetylase activity is essential for prolongation of lifespan and delay of cellular senescence [75, 76]. Thus, the ratio between mitochondrial ROS production and NAD+/NADH redox complex needs to be maintained at the levels, to which the cellular metabolic capacity can adapt. This regulation can be tissue-specific. Insulin signaling in connection to mitochondrial function has been intensively investigated in liver, muscle, or adipose tissue [65]. However, it has not been studied in this context in BMAT and BMSC. We have recently reported that enhanced insulin signaling in BMSC of obese subjects leads to increased OXPHOS activity accompanied with higher ROS production [22••]. We suggested that the presence of this hypermetabolic status of BMSC leads to accelerated senescence phenotype and consequently impairment of stem cell functions and increased risk of bone fragility in obesity and diabetes (depicted in Fig. 1).

## Perspectives

Obesity and T2D are increasingly recognized as risk factor for bone fractures [77–81]. New therapeutic strategies are needed based on understanding the biological and molecular mechanisms of BMAT formation. Thus, unraveling the relationship between BMAT and bone metabolism at the molecular level is relevant.

Based on our understanding of insulin signaling and BMAT formation, it is possible that inhibiting insulin signaling within BMSC may serve as a protective mechanism against expansion of BMAT. This notion is supported by experience of using TZD in treatment of T2D. These drugs increase insulin sensitivity but led to increased BMAT formation and increased risk for fractures [77, 80, 81].

Other approach is metabolic slowing induced by caloric restriction or nutrient supplementation that can prevent accumulation of unused intermediates from metabolic processes and support mitochondrial functions in metabolically active tissues [82–84]. On the other hand, calorie restriction in animals is associated with increased BMAT formation, but its effects on humans remain to be determined.

Given the interactions among medications and lifestyle modifications/interventions, the relative effect on BMAT metabolic phenotype and insulin signaling within bone microenvironment needs studying to identify specific approaches for prevention and treatment of metabolic bone diseases associated with obesity and T2D.
